# #JusticeforGeorgeFloyd: How Instagram facilitated the 2020 Black Lives Matter protests

**DOI:** 10.1371/journal.pone.0277864

**Published:** 2022-12-07

**Authors:** Ho-Chun Herbert Chang, Allissa Richardson, Emilio Ferrara

**Affiliations:** 1 Annenberg School for Communication and Journalism, University of Southern California, Los Angeles, CA, United States of America; 2 Department of Computer Science, University of Southern California, Los Angeles, CA, United States of America; 3 Information Science Institute, Marinal del Rey, CA, United States of America; University of Vermont, UNITED STATES

## Abstract

We present and analyze a database of 1.13 million public Instagram posts during the Black Lives Matter protests of 2020, which erupted in response to George Floyd’s public murder by police on May 25. Our aim is to understand the growing role of visual media, focusing on a) the emergent opinion leaders and b) the subsequent press concerns regarding frames of legitimacy. We perform a comprehensive view of the spatial (where) and temporal (when) dynamics, the visual and textual content (what), and the user communities (who) that drove the social movement on Instagram. Results reveal the emergence of non-institutional opinion leaders such as meme groups, independent journalists, and fashion magazines, which contrasts with the institutionally reinforcing nature of Twitter. Visual analysis of 1.69 million photos show symbols of injustice are the most viral coverage, and moreover, actual protest coverage is framed positively, in contrast with combatant frames traditionally found from legacy media. Together, these factors helped facilitate the online movement through three phases, culminating with online international solidarity in #BlackOutTuesday. Through this case study, we demonstrate the precarious nature of protest journalism, and how content creators, journalists, and everyday users co-evolved with social media to shape one of America’s largest-ever human rights movements.

## Introduction

On May 25, 2020, a 17-year-old girl named Darnella Frazier used her smartphone to film Minneapolis police officer Derek Chauvin kneeling on the neck of George Floyd for more than nine minutes [1]. She uploaded the footage of the fatal incident to Facebook. Within two days, the video went viral, sparking global outcry. By June 2, 2020, Brianna Agyemang and Jamila Thomas, two Black women and music executives at Atlantic Records, created the viral hashtag #TheShowMustBePaused [2]. They called on recording artists to use their platforms to draw attention to systemic racism by posting a single Black square on their Facebook and Instagram timelines. Millions of everyday people joined the initiative and did the same in what came to be known as #BlackOutTuesday [3].

This study provides a snapshot of the Instagram activity leading up to this viral moment, tracking it through its apex and eventual nadir. Our investigation is novel, insofar as #BlackOutTuesday likely marks the first time in the history of social media movements that a visual platform—not a predominantly text-based one like Twitter—took center stage. Twitter has been well-studied since the Arab Spring uprisings of 2011 [4–7]. Most of these investigations have focused on textual diffusion, however, ignoring the adage that a picture is worth a thousand words. In modern social movements, it is often an incendiary photo or video that galvanizes the public’s support for a social justice issue [8–11]. These images can seem fleeting, however, because ephemeral platforms such as Instagram feature videos that disappear after 24 hours and an endless scroll of content that can be hard to search. This study, therefore, answers a recent call for media scholars to document more visual social media content for scholarly analysis [12]. The data collection we carried yielded a corpus of almost 1.7 million photos obtained during the June 2020 Black Lives Matter protests.

Our review of prior literature features three parts. First, we explain how (and why) Twitter was invaluable to the first wave of the Black Lives Matter movement, while its second wave shifted toward Instagram, according to extensive qualitative work. We then summarize the evolution of Instagram studies to emphasize further why we chose to highlight this emergent site for protest journalism. Lastly, we consider the press concerns of protests and the broader theory of connective action, namely how modality and affordances impact frames of legitimacy and the emergent opinion leaders. Through this frame, our results demonstrate the precarious nature of movement media, and how content creators, journalists, and everyday Instagram users co-evolved their earlier social media practices to report on and shape one of America’s largest-ever human rights movements.

### Twitter and the first wave of Black Lives Matter

The Black Lives Matter movement began in 2013, when George Zimmerman was acquitted for shooting and killing Trayvon Martin, an unarmed Black teenager, in Florida. Alicia Garza, a well-known political organizer from Oakland, California, wrote a love letter to Black people after the verdict was announced and posted it on Facebook [13]. She ended it with the sentence, “Black Lives Matter.” Her friend and fellow organizer, Patrisse Cullors, added a hashtag to the front of the message and re-broadcast it on Twitter. It would be one more year before the #BlackLivesMatter hashtag went viral in 2014. On August 9, a White police officer, Darren Wilson, shot and killed an unarmed Black teenager, Mike Brown, in Ferguson, Missouri. When police allowed Brown’s body to lay in the road for four hours, uncovered in the sweltering summer heat, peaceful protests began [14]. Citizen journalists used the hashtag to document Brown’s killing and the community’s outrage [15]. Activists used the #BlackLivesMatter hashtag to call on the police department to cover up Brown out of respect for his humanity. For the rest of the summer, use of the hashtag soared [16].

Groups that opposed the new Black Lives Matter movement created counternarrative hashtags, such as #AllLivesMatter (to reject claims of racism in policing) and #BlueLivesMatter (to support law enforcement) [17–19]. An exhaustive study of Twitter discourse found that six major communities relied on Twitter to discuss police brutality in 2014 and 2015: Black Lives Matter activists, Black entertainers, conservatives, bipartisan reporters, legacy media outlets, and young Black Twitter users. Freelon, McIlwain, and Clark (2016) analyzed 40.8 million tweets, more than 100,000 web links, and 40 interviews with frontline activists and allies, and discovered that the vast majority of those who tweeted using the #BlackLivesMatter hashtag denounced police brutality [20]. The authors also found that activists who tweeted movement-related news succeeded in educating “casual observers” who either expressed “awe and disbelief at the violent police reactions to the Ferguson protests” or “conservative admissions of police brutality,” especially in the cases of Eric Garner and Walter Scott’s public police killings. Overall, the research postulated that activists’ primary goals for using Twitter were “education, amplification of marginalized voices, and structural police reform”.

Subsequent inquiries into the Black Lives Matter movement’s use of Twitter in 2016 included how users craft counternarratives to anti-Black racism [12, 21], how Black feminists hijacked the platform to highlight intersectional struggles [21, 22], and even how Millennial organizers were departing from the Civil Rights Movement’s protest templates to create their own [23–25]. The Pew Center, for example, reported in 2016 that the #BlackLivesMatter hashtag peaked during the 10 days spanning July 7-17—with nearly 500,000 tweets of the back-to-back killings of Alton Sterlingaily. Studies such as these centered around Twitter as a digital public sphere [26]. In the book Hashtag Activism, for example, the authors argue that the Black Lives Matter movement, and other contemporary Twitter-based movements then, were propelled by strong Twitter communities of Black women. The #GirlLikeUs transgender justice campaign, or the #SayHerName movement to raise awareness about Black women victims of police brutality, flourished and thrived on the platform [27]. As the decade came to a close, the Black Lives Matter movement waned. Although publications such as The New York Times or The Atlantic published end-of-decade pieces on how social media shaped modern protest in the 2010s, the emphasis remained on Twitter’s impact—until this study.

### Instagram and the second wave of Black Lives Matter

In May 2020, Darnella Frazier’s cellphone video of George Floyd’s murder reinvigorated the Black Lives Matter movement—in the same way that the photographs of Emmett Till’s 1955 lynching rebooted the dwindling, post-World War II Civil Rights Movement [28]. In Bearing Witness While Black, Richardson draws these parallels, arguing that pictures and videos have played an outsized role in Black movement-building. Richardson also documents the rise in smartphone video as a tool for political testimony to explain why Black Americans “press record” as a means to fight back against systemic oppression in the US [12].

Media pundits and scholars have in recent years begun exploring Instagram’s affordances, and early content largely fell outside of the scope of social movement media. An early probe into the world of Instagram endeavored to explain how users’ “experiences of production, sharing, and interaction with the media they create” are mediated by the “interfaces of particular social media platforms” [29]. This study was one of the first to use computational analysis and visualizations to explore Instagram’s social and cultural patterns. The team compared the visual signatures of 13 different global cities using 2.3 million Instagram photos, and honed in on 200,000 Instagram photos that were uploaded in Tel Aviv, Israel. While the three-month study confirmed that one could ascertain people’s activities and political habits from the geotagged photos, there was not a sustained look at a particular viral moment.

Other Instagram studies from the 2010s followed a similar pattern. Scholars often investigated what images people uploaded to Instagram [30] or how to detect the age of a user from their photos [31], but stopped short of analyzing Instagram photos during moments of political unrest. Still other studies elucidated the effects of pop culture [32], or the five primary social and psychological motives of Instagram use: “social interaction, archiving, self-expression, escapism, and peeking” [33]. Recent studies on political movements followed en suite. Meme pages and celebrities, for instance, have been recently characterized to spark instances of political participation in Morocco [34], maintain partisan identity in Canada [35], promote public health behavior [36], or facilitate misinformation [37]. Recent work has focused on the growing role of visuals in protests directly, such as the case of the Hong Kong Protests in 2019 [38]. Amid the virality of the #BlackOutTuesday hashtag on Instagram we decided to study the visual platform, finally, in a political context.

The backdrop of the global discourse about Mr. Floyd’s controversial video was not the only reason for the shift away from Twitter and toward Instagram. Instagram content is four times more likely to be geotagged than Twitter content, which provides us invaluable insight into when and where the #BlackOutTuesday groundswell occurred. Instagram provided the right lens for studying the visual dimension of this phenomenon. Recent studies have also shown the importance of racial presentation in mediating (mis)information dissemination [39], which may be more evident in the visual mode. Furthermore, the Twitter discourse we analyzed seemed more like “ambient journalism” [28] that was always on, rather than the more intentional educational and call-to-action-style posts that we observed casually on Instagram.

Lastly, we shifted our focus toward Instagram because the people did too. Many of the #BlackOutTuesday posts on Twitter linked back to an original post on Instagram, using either Facebook’s native algorithmic syncing tool or the IFTTT (IfThisThenThat) application. We were also intrigued by Instagram’s many technological affordances. The platform remains a walled garden, which does not make it easy for users to hyperlink outward. That is, Twitter can reference Instagram posts but not vice versa, creating an asymmetry in information flow. Trapping captive audiences within Instagram gives the platform enormous power over what the user sees—in that content is mediated solely through one platform. Without trigger warnings or decency screens over controversial content, Instagram users in June 2020 faced an increased likelihood of viewing potentially harmful content while scrolling through their feeds. Likewise, users found that they were beholden also to Instagram’s opaque user guidelines around protest posts. A USA Today piece reported that Black users were suspended from Facebook for even talking about racism [40]. Similarly, Instagram and TikTok apologized for algorithmically silencing Black voices [41, 42].

Our last preoccupation with Instagram during the #BlackOutTuseday campaign centered around protesters who were captured in the viral pictures. Their visibility meant that their chances of being added to police’s facial recognition databases or other forms of AI-based cataloguing grew. Much like the 2019 Anti-Extradition Law protests in Hong Kong—when activists combatted doxxing by communicating through an high-level encryption app, Telegram—we observed users creating 24-hour ephemeral Instagram “Stories” that were designed to disappear, presumably to limit the retrieval of their movement media.

There is certainly the perspective that #BlackOutTuesday was performative and did not directly advance the movement. For instance, a recent study found, through 20 interviews of wellness influencers, that sharing of the square was for maintaining credibility with their following base [43]. However, their work focuses primarily on influencers, users that have achieved a certain level of popularity, and in wellness, which is an even more specific slice of Instagram. Moreover, the greater point is that there was sufficient demand for influencers to navigate the movement, which creates a larger presence on Instagram than previously, whether solidarity is performative or not.

### Organizational dynamics: Modality and opinion leadership

Modality, taken from semiotics, refers to the format for which information is stored, prior to presentation. The difference between Instagram and Twitter is thus the visual and textual mode, respectively. As such, Instagram is poised to advance research on how visuals aid social movements when held in comparison with the textual mode. We are particularly interested in two dimensions: framing and opinion leadership. First, the visual modality of engagement—through photos and video— demands a different form of participation than through text. Per the adage “a picture contains a thousand words,” visuals are dense and powerful message holders. Studies have shown visual messages are more temporally efficient [44, 45] and amplify the affective reaction and reinforce textual content [46, 47]. Beyond photos, injustice symbols can serve as a force that extends political efforts beyond local, even national, accounts [48].

More critically, this form of engagement bears many similarities to the traditional relationship between press and protest. Long-standing work has shown institutional news media portrayals delegitimize collective action [49–51]. As conceptualized, images of protest are distributed by legacy media sources. Protestors in turn sought their attention through disruptive or combative tactics, which led to condemnation, and thus delegitimization. The participation of citizen journalists, theoretically, would remove this necessity, and recent scholarship explores this possibility. A recent experimental study finds visual frames important for increasing support and identification toward protesters [52].

Apart from the modality itself, the legacy media has traditionally occupied opinion leadership, and this flow of information through elites has garnered significant attention since the proposition of two-step flow from more than half a century ago [53]. While research that intersect visuals in social movements on digital media remains sparse compared to textual analysis, there are still numerous studies. For instance, Neumayer and Rossi (2018) analyze images recently studied photos and videos of Twitter during the Blockupy protests in Frankfurt [11]. However, they found reinforcement in regard to the politics of visibility, where institutional and official accounts still garnered the most attention (retweets). Instagram, with its less-public interface and limited hyper-linkage could poise as a counterpoint to Twitter’s organizational dynamics.

### Research questions

While offline events–such as widespread protests–certainly also occurred, contemporary social movements are coordinated significantly through online spaces, such as social media. Various reports have highlighted the importance of Instagram. If our assumption that a) online social platforms are important to protest organizing and b) Instagram has taken an outsized role compared to prior movements and Twitter, then answers to these questions can address how solidarity can be in part attributed to shifts in framing and shift in opinion leaders found on Instagram.

As such, our investigation into Instagram offers rich comparative insight toward a) how the modality of engagement shifts frames of legitimacy, and b) the nature and role of emergent opinion leaders. We offer the following research questions and hypotheses:

1. **Characterization**: What were the temporal trends of June 2020’s second wave of the Black Lives Matter movement, in terms of frequency, geography, and textual content?
2. **Injustice Symbols and Legitimization**: What were the top shared images on Instagram, and how do they semantically, affectively, and symbolically function?
3. **Network and Opinion Leaders**: Do the central actors and communities that emerged on Instagram reinforce the institutional media, or are they conducive to grassroots connective action?

## Materials and methods

### Data collection and description

We monitored Instagram posts for #JusticeForGeorgeFloyd from May 28, 2020 to June 30, 2020, extracting the top shortcodes from the public hashtag page (every two hours using the *Instaloader* package). The hashtag was chosen as it was the top trending hashtag on Twitter, Instagram, and Facebook from the three days prior. Posts were then extracted through static short codes, including all photos, videos, post-specific public metadata, and comments. No personal information was collected, and non-verified accounts were hashed. Data was collected using Instagram’s public page with public posts only. The entire dataset consists of 1,147,278 posts and descriptions and 1,694,909 photos.

We found 155,282 of 1.13 million culled posts (13.7%) have location tags. This is a significantly higher rate of geotagging than in a Twitter dataset, which averages 3-4% geotagged posts [54–56]. We posit this arises due to the visual, scrapbook nature of the platform, as pictures taken live are associated with a physical location. The inclusion of this metadata allows us to understand the flow of protest geographically with much higher statistical power. Table 1 shows the total number of posts by state, overviewing the distribution of US-based participation. While California, Florida, New York, and Texas occupy top positions due to their large population, Minnesota is significant since the movement originated from there. Washington, DC generated a high level of participation as well, relative to its population.

**Table 1:** Top states by number of posts and comparison with actual population statistics. The percentage by population shows the actual percentage relative to the entire population of the United States. As such, the post-to-pop. ratio describes the level of over-representation a certain state has, with D.C. leading at 20.14 times the representation.

State	# of Posts	% of posts	Pop. Rank	% by Pop	Post-to-pop. Ratio
CA	32,705	21.10%	1	11.91%	1.77
NY	23,477	15.10%	4	5.86%	2.58
MN	14,935	9.62%	22	1.70%	5.66
TX	10,212	6.57%	2	8.74%	0.75
FL	7,501	4.83%	3	6.47%	0.75
DC	6,573	4.23%	49	0.21%	**20.14**
GA	6,506	4.19%	8	3.20%	1.31
IL	4,704	3.03%	5	3.86%	0.78
PA	4,685	3.02%	6	3.82%	0.79
OR	3,068	1.98%	27	1.27%	1.56

To discern the discursive dimension of the protest, we collected the top hashtags and sorted them by usage in Table 2. These include iterations of George Floyd (such as #JusticeForGeorgeFloyd and #GeorgeFloyd) and the Black Lives Matter movement (#blm and #blacklivesmatter). The phrase #icantbreathe also emerged as an important hashtag, co-occurring in 10% of all posts.

**Table 2:** Top hashtags of 540,591 unique hashtags.

Hashtag	counts	%	hashtag	counts	%
justiceforgeorgefloyd	1,147,278	100%	minneapolis	43,066	4%
blacklivesmatter	719,046	63%	equality	42,539	4%
georgefloyd	301,076	26%	stopracism	38,684	3%
blm	224,415	20%	peace	37,484	3%
blackouttuesday	198,372	17%	breonnataylor	36,603	3%
justiceforbreonnataylor	156,208	14%	black	35,466	3%
justiceforahmaud	144,287	13%	repost	34,324	3%
icantbreathe	126,407	11%	alllivesmatter	32,894	3%
justiceforfloyd	122,818	11%	saytheirnames	32,607	3%
nojusticenopeace	114,406	10%	blackoutday2020	31,062	3%
justice	109,319	10%	justiceforahmaudarbery	30,918	3%
protest	85,273	7%	ahmaudarbery	28,020	2%
policebrutality	61,884	5%	acab	27,212	2%
racism	54,935	5%	usa	26,973	2%
love	53,351	5%	endracism	26,849	2%

Moreover, the important feature of this second-wave Black Lives Matter movement was that it demanded legal and moral justice for other people who died from white supremacist vigilantism and police brutality around the same time as Mr. Floyd. Dual campaigns for Ahmaud Arbery (who was killed by three White men in Georgia that spring while jogging) and Breonna Taylor (whom police killed in Kentucky after issuing a mistaken no-knock warrant to her home) also emerged. The #saytheirnames hashtag attempted to connect these cases.

Briefly, the #JusticeForGeorgeFloyd hashtags occurred prior to his death. A quick survey indicated these hashtags were added retroactively to posts to generate attention and traffic, what scholars might refer to as the pursuit of clout. These posts were filtered out.

### Visual content analysis through perceptual hashing

To determine the top visual content that emerged from the movement, we first identified similar figures by conducting a perceptual hash (p-hash) on each image. This converts each picture into a 64-bit string, which is then used to extract the similarity between photos and identify the most popular images based on its hashes (Zauner, 2010). The algorithm then reduces image (usually to 32 x 32 pixels), then applies greyscaling, cosine transformation, then reconversion to a string, and thus yielding the resultant p-hash.

### Network analysis

We then constructed an interaction network using the full set of Instagram comments. A network is a set of nodes, which are connected by a set of edges. Edges can be directed or undirected. Directed nodes indicate a directional relationship (such as unreciprocated following relations on Twitter) or undirected (such as friendships on Facebook). In our analysis, direct edges are constructed between Instagram posters (source node) and people who comment (destination node). Additionally, associated with each edge is a weight—the frequency of times two users interact in the comments section. We observe users who interact hundreds of times within their comments in such a short timespan of 30 days. This resulting network contains 3,337,890 unique users and 3,976,914 unique edges.

We then aggregated these networks into state-level networks, to better understand how communication flowed inside and outside local communities. We describe this formally below. Let *U* denote the set of users and S represent the set of States. *A* represented the adjacency matrix for users (columns) and States (rows). We then aggregate *A* into a State-level adjacency matrix. Let *i* be the row index and *j* be the column index (*a*_*i*,*j*_ the element). The algorithm for generating the state-state network can then be summarized in Algorithm 1.

$$\begin{array}{c} A=\text{Adjacency}\;\text{Matrix}\;\text{with}\;\text{Dimensions}\;|U|\times |S|\\ A_{S}=\text{Pre-allocated}\;|S|\times |S|\text{matrix}\\ \text{for}\;\text{i,}\;\text{state}\in \text{enumerate}(S):\\ \;E≔\left[v_{j}\right]\;\forall j\text{s.t.}a_{i,j}>0,v_{j}\text{is}\;\text{a}\;\text{column}\;\text{vector}\\ \;A_{s}\left[i,*\right]={\left[\sum_{j\in E}v_{j}\right]}^{T} \end{array}$$ (1)

In simple terms, for each user (represented by a column), we considered all the geographical sources to which the user had been exposed. If a user had engaged with a post from both New York (NY) and Minnesota (MN), then the incoming edge from MN to NY (denoted MN→ NY) would be equivalent to the number of times the user engaged with content from MN. Vice versa, NY→ MN denotes the number of times the user engaged with posts from NY. The user thus serves as a proxy for cross-engagement between states. The sum of all user-state aggregations is the total weight between two states.

We also share a brief note comparing Instagram and Twitter network construction. Since Instagram does not allow URL sharing and hyperlinks, exposure to a post is strictly mediated by Instagram’s algorithm. There is no direct equivalent of a retweet. Commenting on Instagram is closer to replying on Twitter, which naturally impacts the topology of the Instagram network.

## Results

### Temporal characterization of the movement

**Fig 1:**
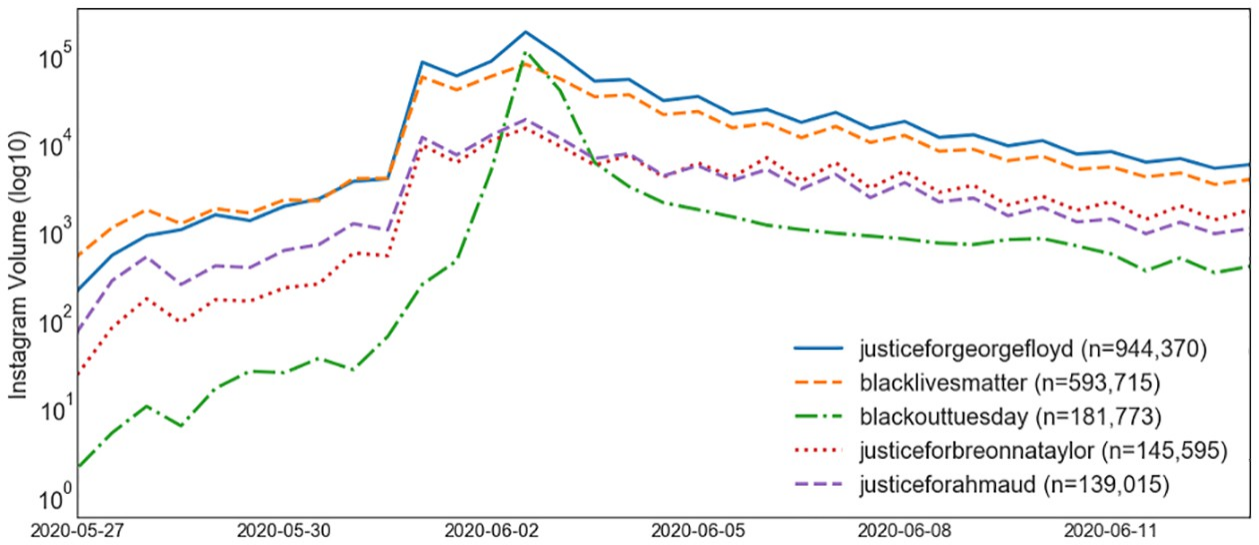
Volume of Instagram posts plotted on an hourly basis, separated by top hashtags shared during the George Floyd protests.

Our first research question concerns what the temporal trends of June 2020’s second wave of the Black Lives Matter movement were, in terms of frequency, geography, and textual content. To address this question, we considered the frequency of posts over the month of protest. Fig 1 shows the distribution of post frequencies on an hourly basis between May 25, 2020 and June 25, 2020, on a log-basis. Right after the killing of George Floyd on May 25, we observed a lull of activity. This was punctuated then by an increase on May 31. This remained consistent until exploding in volume on June 3. Its use then decayed exponentially (from the order of 10,000 to 1,000 in two days).

Fig 1 shows the temporal behavior of top hashtags (as given in Table 2), and shows what generated the spikes of activity between May 31 and June 3. Hashtags can be summarized as a few distinct categories: 1) mentions of George Floyd, Breonna Taylor, and Ahmaud Arbery, and 2) the BLM movement and the slogan #icantbreathe. The one exception is #BlackOutTuesday. It was localized during the spike on June 2, which suggests that the musician-led movement was not just a critical driver of momentum, but the most viral event on Instagram between May 30 and June 3. We can thus split the second wave of the Black Lives Matter movement into two periods: the first, driven organically after the death of George Floyd, and the second, driven purposefully via #BlackOutTuesday event.

#### Geographic network analysis

As noted in the methods, New York, California, and Minnesota claimed the highest activity, which is reflected temporally. We thus our attention to the interaction between various states, to understand the localization of movements, summarized in Table 3. The summary technique follows network flow based on the direction of edges [57–59]. By looking at the circulation of information in, out, and within a state, we can better understand the consumption and attention dynamics from a supply and demand perspective.

**Table 3:** Interstate network structure and proportion of posts seen within-state, exported to other states, and imported from other states.

**Top Self-Flow**				
**State**	**% within State**	**% exported**	**% imported**	**Total posts from state**
SD	81%	11%	8%	447
VT	69%	18%	13%	474
ID	64%	20%	16%	1,760
CA	62%	16%	23%	358,948
WY	60%	21%	18%	179
MN	59%	20%	21%	158,753
**Top exporters**				
**State**	**% within State**	**% exported**	**% imported**	**Total posts from state**
WV	25%	60%	15%	965
NE	36%	50%	14%	1,968
ND	35%	47%	18%	603
NH	37%	44%	20%	1,471
MS	31%	43%	26%	2,495
**Top Importers**				
**State**	**% within State**	**% exported**	**% imported**	**Total posts from state**
DC	43%	28%	29%	83,710
MD	42%	29%	29%	20,912
IA	33%	39%	28%	3,052
NY	55%	18%	27%	268,169
MI	39%	35%	26%	19,452

High self-flow (*SF*) indicates significant localization of the movement, and relatively less attention to other locations. High out-flow (*OF*; exports) demonstrates that a state receives attention from other states. Higher in-flow (*IF*; imports) indicate a state pays attention to other states. In other words, this table shows the supply and demand dynamics at the state-level: if *IF* > *OF*, then demand outstripes supply. If *OF* > *IF*, then supply outstrips demand. We choose this state-level normalization as otherwise, small states will be lost in the analysis.

Immediately, we noticed a high percentage within several states, with South Dakota at 0.805 and Vermont at 0.688. California had the highest frequency of posts at 0.617 (rank 4) and Minnesota at 0.589 (rank 6). We reached two conclusions. For states with high levels of participation (CA and MN), we observe high levels of self-generated content within the state. Interestingly, engagement with local activism is similar for states with smaller volumes of participation, which we posit may be due to their relative isolation.

We contrasted this to States with comparatively high levels of content “exports” and “imports.” What we mean by exports is the proportion of posts engaged by users connected to other States. What we mean by imports is the proportion of posts users engaged with that hailed from other States. Washington, DC is the highest importer, indicating the region had limited amounts of self-generated content. While DC is politically active, they also have a smaller population. Because small states have less people compared to externally. These results suggests for small states, connective action is demand-driven. Smaller states will feel like part of a bigger whole, because most their interaction occurs from outside. For the largest states, there is significant self-content. New York is also a top importer. However, it also boasted high levels of engagement within its own State, indicating high levels of participation. As such, east coast centers, while having considerable self-content, also pays attention to what is happening to other states.

**Fig 2:**
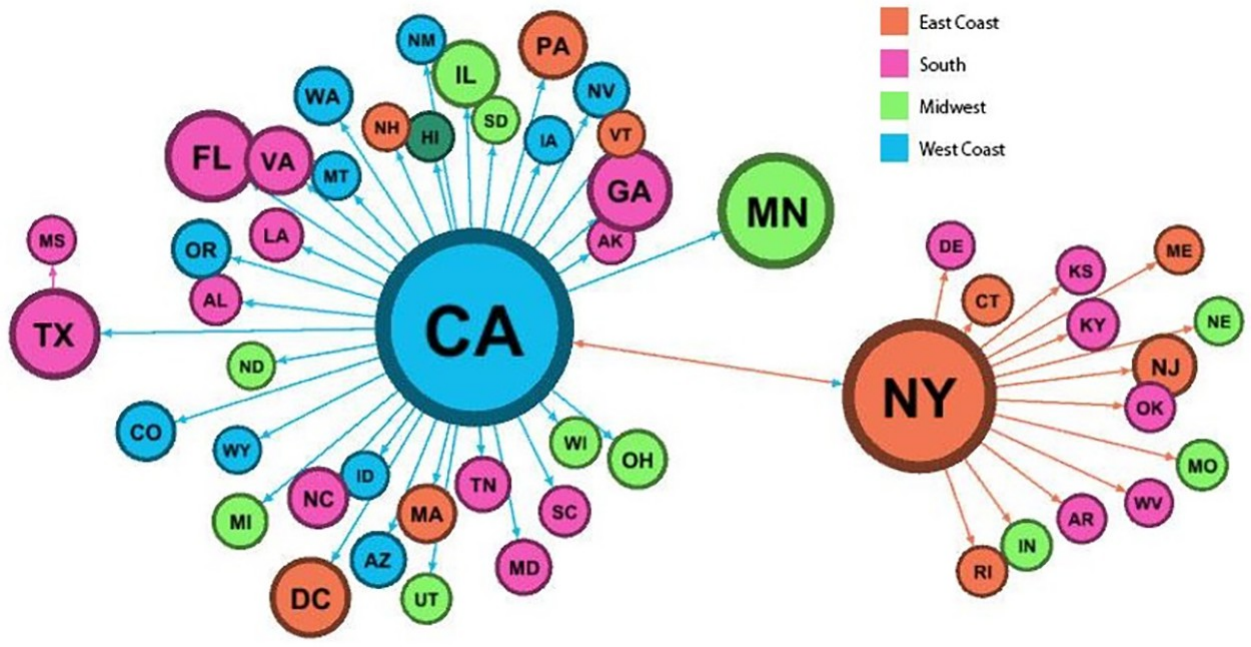
Network of state-state exposure based on aggregated user engagement. Direction of attention are shown by arrows, then colored by general regions in the United States.

We show these dependencies further in Fig 2, which each state’s top destination based on all user engagement. We observe three epicenters: California, New York, and Minnesota. California received the most content from New York and, reciprocally, New York from California. New York seemed to hold the attention of East Coast states, such as New Jersey, Maine, Rhode Island, and Connecticut, along with Southern States closer to the East Coast, such as West Virginia. The rest of the states, except for Mississippi, co-occurred most frequently with California. Mississippi co-occurred most frequently with Texas. These results harken back to prior US-based case-studies. During Occupy Wall Street, observed a similar hub-spoke structure on Twitter, although its activity was localized in California, New York, and Washington DC [60].

To summarize, we identify two interesting trends. First, the time series suggests we break the movement into two waves, starting from May 31 and June 3 respectively. Second, while we observe two epicenters in New York and California, we also observe highly localized, generative activity in Minnesota and smaller states, with geography-based correlations. These would suggest different extents for which messages are propagated [48]. Thus, to understand this dynamic more clearly, we now move into the critical parts of our analysis—visual content and network analysis.

### Injustice symbols and legitimization

#### Visual content analysis

**Fig 3:**
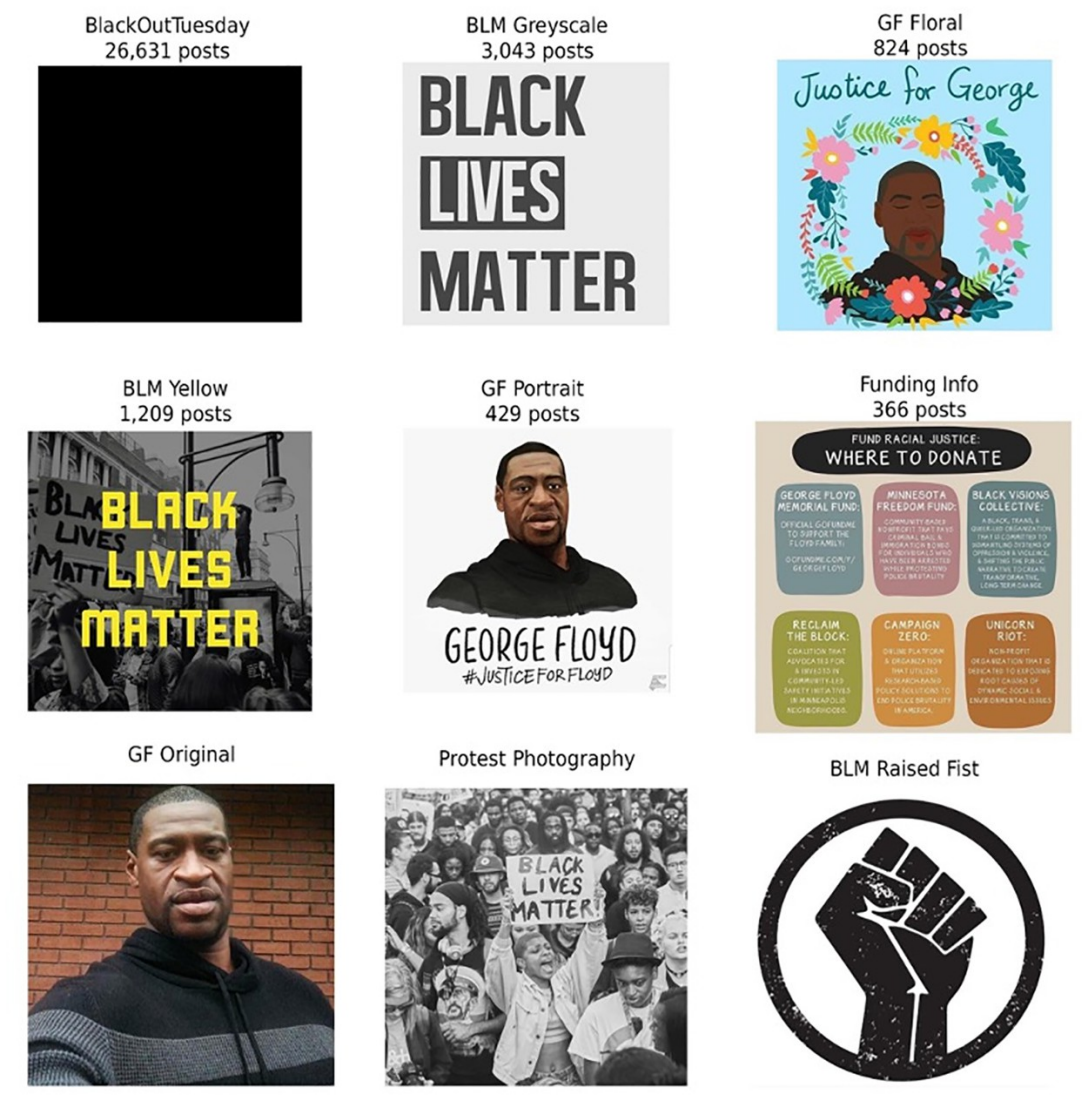
Top photos that emerged from the 2020 George Floyd protests. From top to bottom and left to right, we have the Black Out Tuesday Square (a), logos and icons of the Black Lives Matter movement (b, d, i), portraits of George Floyd (c, e, g), and photos of protest (h).

The focus of this section are the top shared images on Instagram, and how they semantically, affectively, and symbolically functioned. Fig 3 shows the most shared photos during the month of protest. We see a few themes, which we used to construct a typology. The most popular photo during the period we observed was the #BlackOutTuesday full black square. The second type of popular posts were three iterations of George Floyd’s portrait. The first is his original selfie (GF original), a stenciled style (GF portrait), and a floral version in remembrance (GF floral). The third type of common posts were official logos for the BLM movement, which we denoted as BLM grayscale and BLM yellow. The fourth type of common posts were the edited pictures of protest, such as the one in the bottom center. Note, these were prevalent further down the list of top photos. Lastly, the fifth common post type focused on information sharing and organizing, such as the infographic found in the center left. This infographic contains six places to donate money, such as foundations, bail for protestors, or medical fees for those harmed during the protests.

Per our discussion of injustice symbols, none are as iconic as the raised fist, which was among the top 9 most diffused icons. And in general, 7 of the top 9 are all abstractions or calls to a greater movement. For instance, the most popular of Mr. Floyd’s portraits is the floral rendition—one that emphasizes memorial. As previously theorized for the world before social media, while protesters would complain about being misconstrued as angry and combatant, this was one of the only ways of gaining media attention and thereby generate awareness through cable television [49, 61].

Instead, in this Instagram-led protest, coverage of the protest seems to have come secondary to these injustice symbols. Furthermore, the logic that brings about this coverage of protest action is based on the algorithm, and thus grassroots popularity. The effects of this change in logic is most clear with how protesters are framed. Instead of the deviant protesters, we see poignant, positive frames of protesters in action.

How do these photos act together? From a rudimentary level, these photos resemble prior typologies of protest content based on Twitter [62]: (1) political mobilization; (2) coordination; (3) information; and (4) conversation. However, unlike Tweets, these individual photos resist singular placement into these four categories. The #BlackOutTuesday square is simultaneously a call for action, and a form of coordination to incite conversation. Similarly, the George Floyd portrait is political mobilization, information, and conversation, as its form shifts over time. Early forms of George Floyd’s portrait were more targeted toward information sharing, whereas latter ones were for conversation. Only the one for funding sources has an explicit informational categorization.

Here, we experience a distinction with theories based on Twitter. Whereas prior classification is based upon clear, textual information—the four-pronged typology cannot be applied neatly to images, since the categorization depends more on how it is used (the coordinated flooding of images) and its actual contents (as part of a broader piece of social justice discourse). Note, while coordinated flooding has happened on Twitter [36, 63], this has been primarily textual.

**Fig 4:**
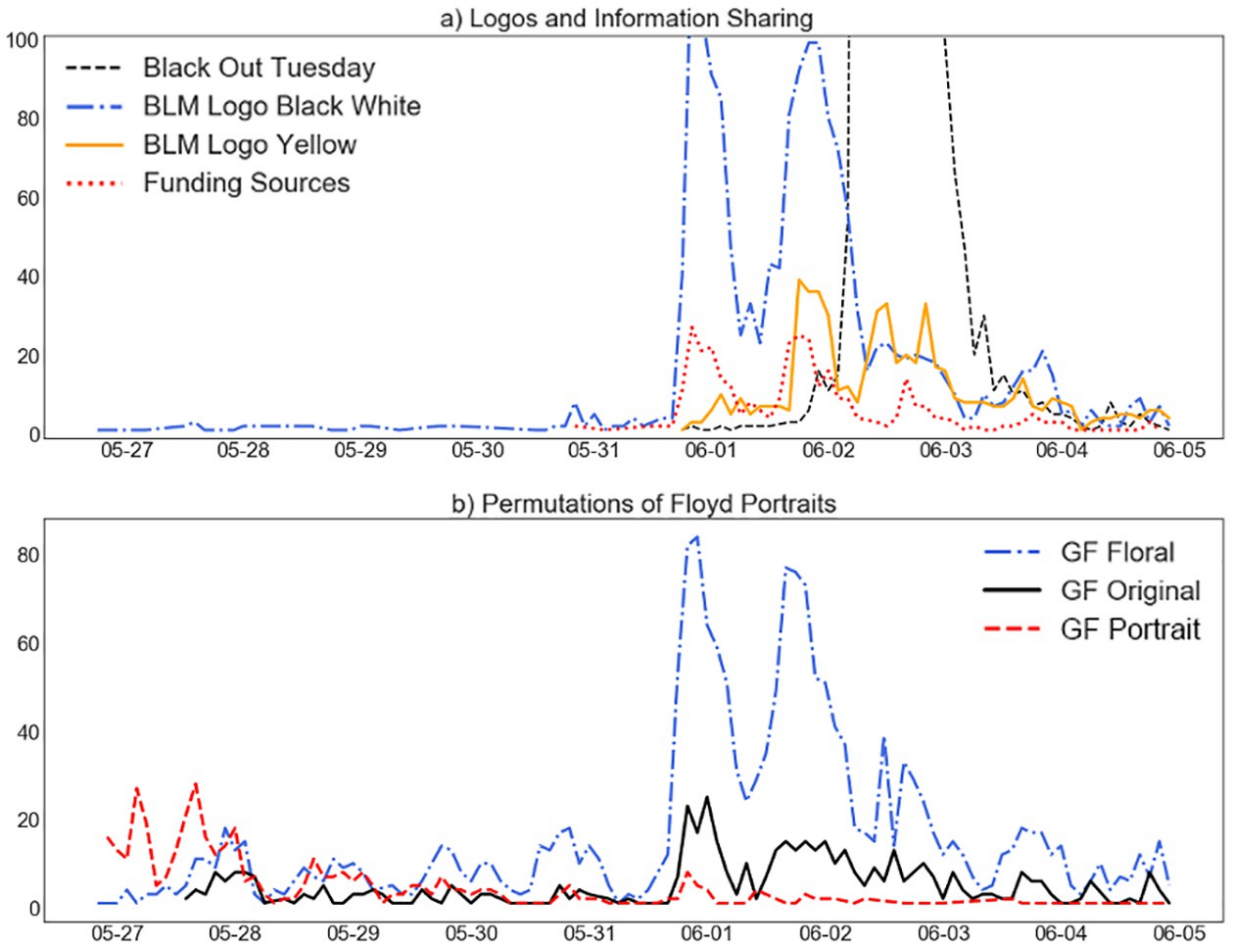
Time series of top icons diffused during the George Floyd protests. Fig 4a) shows the diffusion of the three BLM logos and the funding infographic. Fig 4b) shows the three iterations of George Floyd’s portrait. We observe much earlier volume in the portraits as compared to BLM and protest organizations.

We can further analyze the evolution of these photos temporally. Fig 4 shows the BLM logos, the full black squares of #BlackOutTuesday, and the funding source. Before May 31, there was little volume from the BLM logos, until the grayscale logo exploded in volume—along with information about funding. This indicates some form of coordination (whether organic or pre-determined). Then, on June 2, (the day of #BlackOutTuesday), another eruption of posts emerged. We observed these beginning as early as midday on June 1, yet the small notch in the black line right before June 2 suggested that users waited for the actual campaign day before posting.

Fig 4b) shows the permutations of the portraits of George Floyd. Notably, the sharing of these photos began much earlier than the BLM logos. The first post that emerged at scale was the realistic portrait, rather than the stylized, floral version of him. Then, on the night of May 31, there was a great acceleration of his floral portrait. The popularity of his floral portrait, designed in remembrance rather than simple outrage, became the most popular icon involving him as a person. Similar portraits of remembrance were made for Breonna Taylor. In other words, the shift in portrait preference also indicates a shift from mobilization and information to conversation and memorial.

By comparing panels a) and b), we observe an early focus on George Floyd as an individual, as there was volume for his portrait rendered early on. The Black Lives Matter logos emerged on May 31, coinciding with the massive protests on the following weekend and thus generating critical mass. We thus make a temporal observation—the death of individual Black Men coalesce once they relate to symbols of injustice.

In summary, by shifting to algorithmic-curated content, one that is based on grassroot popularity rather than institutional agenda setting, the visual frames shift from combatant to memorial, protestors from deviant to poignant. Visual frames also shift overtime, as victims of social injustice are affectively connected to prior symbols of injustice. Next, for us to claim these symbols emerge through algorithmic logic, we need to investigate the central users—namely whether this content was dictated by the institutional media or not.

### Networked flow and opinion leaders

Next, we investigate the central actors and communities that emerged on Instagram. Table 4 aggregates the top 10 accounts by the number of likes. What is most interesting about these top users is that their differences largely outweigh their similarities. The top-ranked user is The Shade Room, an online publication that specializes in celebrity gossip specifically within the Black community, which sets the tone for our focus on entertainment-based groups, specifically meme-related pages. Accounts that fall into this category include thedisappointingexperience, which puts out a large volume of posts, and tenth-ranked DomisLiveNews, which combines world news in a meme format, often with a hyper-focus on trivial events such as a Black rapper changing into a pair of new Yeezy sneakers. Their presence on the list is likely due to their large, pre-existing audience base.

**Table 4:** Top 10 accounts by likes in the dataset. Opinion leaders include magazines and meme pages, in addition to institutional or established celebrities.

Username	Likes	Name	Account Description / Occupation
theshaderoom	7,849,147	The Shade Room	Publication of celebrity gossip predominantly within the Black community
_stak5_	6,405,100	Stephen Jackson, Sr.	Former NBA player
midianinja	3,741,410	midia NINJA	Independent narratives and journalists based in Brazil.
the_female_lead	2,611,844	The Female Lead	Account of an education charity
iamjamiefoxx	2,191,800	Jamie Foxx	African-American actor
thedisappointingexperience	1,816,197	N/A	Semi-automated meme account
gentlemanmodern	1,610,376	Gentleman Modern	Independent photographer based in New York
diet_prada	1,421,634	Diet Prada	A fashion watchdog group that emerged as a serious voice campaigning for integrity and accountability within the industry
shaunking	1,382,424	Shaun King	American writer and civil rights activist
domislivenews	1,372,158	DomisLiveNews	Meme group for hip-hop and world news

Surprisingly, there are only a few real individuals in comparison to the organizations. We observed a former NBA player (Stephen Jackson Jr.), an actor (Jamie Foxx), an activist/writer (Shaun King), and a photographer (Gentleman Modern). Apart from King, most of these users are celebrities that have a high follower count (pre-existing audience). The photographer stands apart as he is not a public figure. His prominence can be attributed to his viral post of two children (one white and one Black) running toward each other and hugging. This lone video garnered more than 1.5 million likes.

On Twitter, Neumayer and Rossi’s found users with institutional support rise to the top, such as politicians, local police forces, and media outlets. Instagram’s slate of citizen journalists, fashion magazines, and celebrities stands in contrast. What this shows is that Instagram can push the content of non-public figures and organizations to the forefront of a movement. His presence is significant as it highlights the role of individual content creators, serving in a similar capacity to citizen journalists. Anyone who participates can become an opinion leader.

Apart from these entertainment-oriented groups, we also observe other social justice-oriented organizations. For instance, *Diet Prada* is a fashion-related watchdog organization that began from the work of two fashion industry professionals and grew into a significant voice in critiquing its business practices including racism. They are most well-known for identifying *Dolce & Gabbana’s* racist ad in Shanghai which features an Asian man struggling to eat Italian food with a chopstick.

Lastly, *midianinja* deserves its own separate mention. Based in Brazil, its website is almost completely in Portuguese. Instead of an individual or an organization, *midianinja* is a collection of journalists, photographers, and media creators focused rather on a theme. The following excerpt appears on their website:

Based on the collaborative logic of production that emerges from the networked society, we connect journalists, photographers, videographers, designers, and enable the exchange of knowledge between those involved (translated).

The presence of a non-American entity in the top percentile of influencers speaks not only to the global reach of the movement, but also to the role that international organizations took in collaborating with US-based journalists and photographers. Rather than individual journalists networking, existing coalitions own their own Instagram account, which instantiates quite directly the concept of “networks as actors” [64].

Opinion leaders exist in every social network. Understanding differences across platforms provide valuable insight to how platform affordances can engender different types of leaders and information environments. Specifically, Neumayer and Rossi find “visuals in political protest in social media reproduces existing hierarchies than challenges them [11].” They find traditional opinion leaders with institutional power in the form of politicians and the local police forces, which share expressions of latent violence in their visuals. In contrast, entertainment organizations. On Twitter, perhaps due to an algorithm that facilitates public diffusion, the leaders are official accounts and opinion leaders. On Instagram, the most popular accounts are fashion magazines, independent journalist, and meme pages—a collection of opinion leaders that represent everyday content creators and citizen journalists.

**Fig 5:**
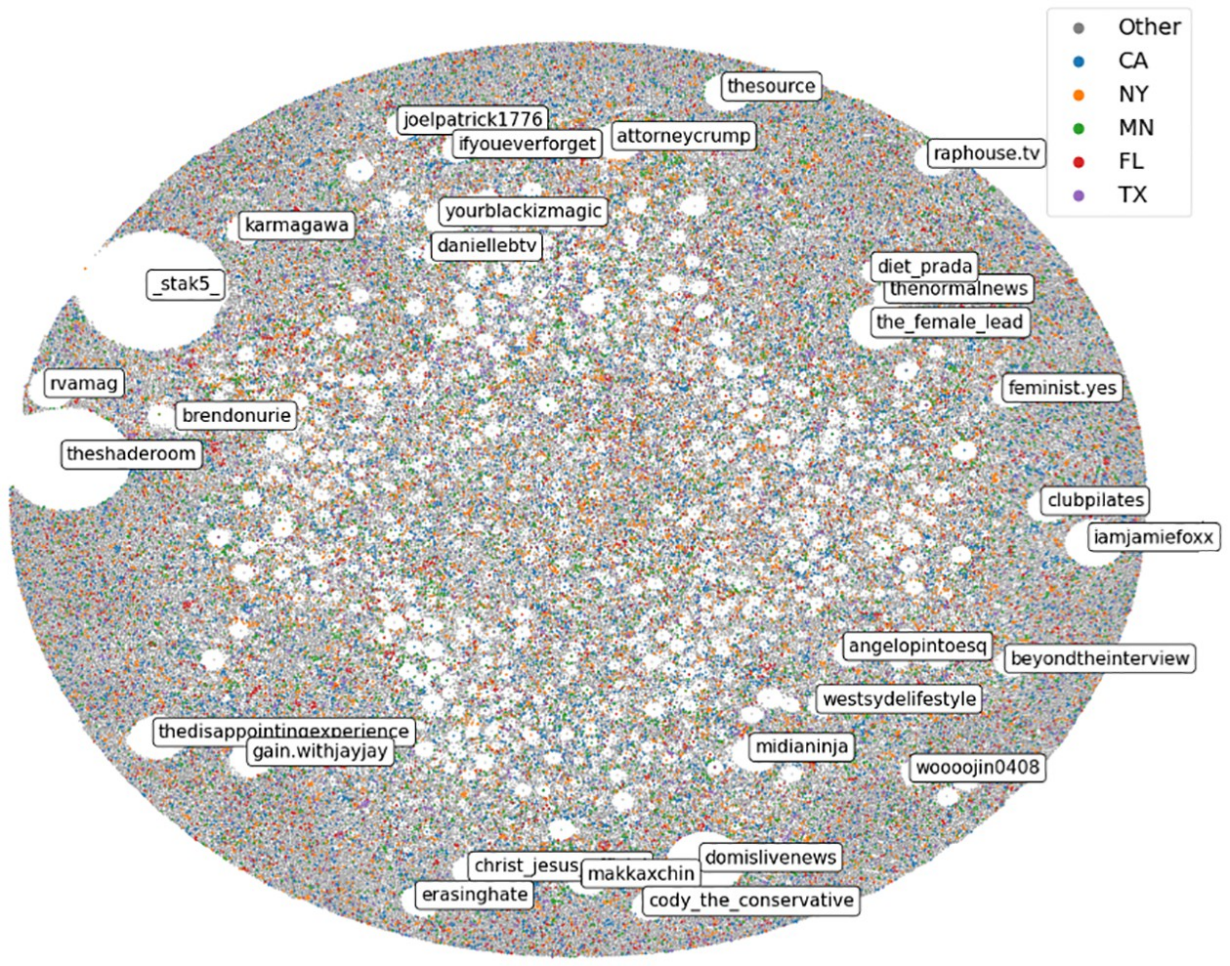
Network of users (n = 713,209), where links are constructed between commenting users and the original poster. Small, dense communities in the center indicate diverse consumption of content.

To drive this comparison home, we close this section with a broad overview of the Instagram public sphere. Fig 5 shows the entire Instagram network, visualized through ForceAtlas, a force-based network layout algorithm [65]. The listed users are the top users by in-degree, meaning ones whose posts featured the most interaction via commenting. The top users are associated with a certain amount of white-space, which corresponds to their level of influence.

One important qualitative observation is the large number of everyday users located in the middle. This indicates a diverse diet from a central core of users, driven by everyday users. Users are also colored by the state for which they consumed the most content. The graph appears heterogeneous (colors are well mixed), which is a testament for its international reach. Most importantly, topologies where users are found clustered in the middle indicate many connected small worlds, which lies in contrast with many Twitter visualizations [55]. On Twitter, direct visualizations lead to “hair-balls”—opinion leaders occupy the center while users retweet or reply to only one of these.

To summarize our answer to RQ3, the most prominent users are non-institutional accounts with existing Black audience-serving publications, individual journalists or content creators, social justice organizations, and meme groups. Unlike Twitter, where preexisting organizations garner more attention, on Instagram entertainers that specialize in content production with an existing audience base generate the most engagement throughout the protests, especially those who have a history of engaging with social issues.

## Conclusion

The purpose of this study was to understand how Instagram mediated the Black Lives Matter protest in 2020, as a counterpoint to Twitter-based research, while intersecting the rich literature on social movements, visual framing, and connective action. We narrow our focus on a) how the modality of engagement shifts frames of legitimacy, and b) the nature and role of emergent opinion leaders who dictate the flow of information. To do so, we analyzed the movement across the spatial, temporal, semantic (both visual and textual), and communal dimensions.

Spatially, three epicenters arose in New York, California, and Minnesota. While the movement garnered large-scale heterogenous reach, localized activity in Minnesota and smaller states emerged. The movement itself consisted of three phases. The first, latent phase arose from the sharing of George Floyd’s portrait following his killing. The second phase began with the explicit mobilization with BLM. The third phase, which generated the largest level of engagement, was from the #BlackOutTuesday campaign. We identified five types of posts: (1) portraits of George Floyd, (2) BLM logos, (3) protest activism, (4) organizational infographics, and (5) the #BlackOutTuesday square. While these post resemble Theocharis’ (2015) characterizations of protest communication, these functions shift as the pictures themselves evolve. For instance, the portrait of George Floyd shifted from informational to means of remembrance. Furthermore, multiple of these functions can fold into a single visual.

With this, we offer a few take-aways. Visuals, when coupled with Instagram’s affordances, allows non-institutional opinion leaders to emerge. This stands in contrast with Neumayer and Rossi’s (2018) study on Twitter, which finds official accounts of pre-existing institutions still holding the most clout [11]. Furthermore, these opinion leaders are independent journalists and entertainment-based accounts, such as content creators and fashion magazines, many that embody the “networks as actors” paradigm described in the logics of connective action [64].

In regard to the modality and images themselves, the iconography evolves as the protest evolve. Moreover, due to the emergence of non-institutional leaders, the visual content of protest differ dramatically. First, instead of actual imagery of protest, the most popular images were symbols of injustice [48]. Second, framing shifts from combatant to memorial, deviant to poignant [49]. Positive framing has the ability to increase support and identification toward protestors, thus gaining legitimacy. Given that social media is the largest source of news, it would not be unrealistic to think most users now obtained most of the protest through platforms like Instagram. Together, these factors that emerged from Instagram’s unique ecosystems helped facilitate the Black Lives Matter movement of 2020.

Our study has some limitations. Since our monitoring is done daily at regular intervals, there may be biases toward the content depending on how Instagram updates its public pages. It is also impossible to tell how Instagram’s filtering algorithm works as well. For instance, we observed that posts by other celebrities were suppressed somewhat on the public page, and more likely to induce a more grassroots perception of the platform instead of reinforcing the presence of popular personas. Additionally, there is the perspective that Instagram and #BlackOutTuesday was a mere instance of hashtag activism. However, regardless of whether such attention was performative or not, this drives traffic disproportionately to Instagram instead of Twitter. As such, the ambient journalism being projected would be distinct from the institutional media or Twitter.

Overall, our research pushes the field forward in a few important ways. Our study confirms observations from recent work: that groups often considered frivolous in their creation and dissemination of content—meme groups and fashion magazines—can spark instances of political participation. Entertainers can get serious, and when they do, they can command the attention and shape the behavior of an entire nation.
